# Serum n-3 polyunsaturated fatty acids and psychological distress in early pregnancy: Adjunct Study of Japan Environment and Children's Study

**DOI:** 10.1038/tp.2016.2

**Published:** 2016-02-16

**Authors:** K Hamazaki, A Harauma, Y Otaka, T Moriguchi, H Inadera

**Affiliations:** 1 Department of Public Health, Faculty of Medicine, University of Toyama, Toyama, Japan; 2 Laboratory for Functional Analysis of Marine Materials, School of Life and Environmental Science, Azabu University, Kanagawa, Japan; 3 Laboratory of Food and Nutritional Science, Department of Food and Life Science, School of Life and Environmental Science, Azabu University, Kanagawa, Japan

## Abstract

N-3 polyunsaturated fatty acids (PUFAs), especially long-chain types such as docosahexaenoic acid, are important nutrients in pregnancy, but the relationship between n-3 PUFA levels and perinatal and postnatal depression remains controversial. This study examined the possible relationship between serum n-3 PUFA levels and psychological distress among expectant mothers in early pregnancy. Data and specimen samples were obtained in a birth cohort study started at Toyama Regional Center in July 2012 as an adjunct study of the Japan Environment and Children's Study. Blood samples were collected at 9–14 weeks' gestation (75% of samples) or after 15 weeks (25%). Subjects with a Kessler Psychological Distress Scale score (K6) ⩾9 were assigned to the psychological distress group (*n=*283). The control group (*n=*283) was matched for age, educational level and family income. Fatty acid composition was determined from serum samples by gas chromatography. Associations between fatty acid levels and incident psychological distress were evaluated by logistic regression. After adjusting for possible confounders, eicosapentaenoic acid showed an inverse association with risk of psychological distress, with an odds ratio of 0.47 (95% confidence interval: 0.30, 0.73) for the highest tertile. This inverse association remained even after applying a higher cutoff score (K6 ⩾13) indicating severe psychological distress (74 pairs). We believe this is the first study to reveal the associations between serum n-3 PUFAs and risk of psychological distress in early pregnancy. Further research is required to verify the causality of these associations.

## Introduction

N-3 polyunsaturated fatty acids (PUFAs), especially long-chain types such as docosahexaenoic acid (DHA), are important nutrients in pregnancy. Because DHA is needed for fetal growth, maternal DHA levels steadily decline after 18 weeks of gestation even under normal dietary conditions.^1^ An animal study has shown that when dams were fed a DHA-deficient diet, DHA from their brain was sourced to ensure a sufficient supply of DHA to the fetus, which served to reduce maternal DHA levels by about 25% from pregnancy to lactation.^2^ In a postmortem brain study of patients with depression and controls, women who had two or more children showed DHA levels decreased by 35% on average compared with women who had one child or no children, regardless of disease state.^3^ Because DHA has such important roles in the brain—for example, in serotonergic neurotransmission, dopaminergic function and regulation of corticotropin-releasing factor, as reviewed by Freeman *et al.*^4^—it has been hypothesized that if low n-3 PUFA levels are associated with depression, one reason women are more vulnerable to depression in pregnancy is due to the depletion in their n-3 fatty acid levels to support fetal growth.^5^

Several epidemiological studies have investigated the relationship between n-3 PUFA levels and antenatal and postnatal depression, but the results remain controversial. Among dietary studies, a large study from the United Kingdom revealed that pregnant women who consumed no n-3 PUFAs from fish were more likely to have high levels of depressive symptoms at 32 weeks of gestation.^6^ However, when the association was examined between n-3 PUFA intake at 32 weeks of gestation and postpartum depression at 2 and 8 months, only postpartum depression at 2 months showed a weak association. In Denmark, a very large prospective birth cohort study showed no association between fish or n-3 PUFA intake during pregnancy and postpartum depression.^7^ In Japan, of three studies investigating the relationship between n-3 PUFA intake and perinatal depression,^8, 9, 10^ one showed beneficial effects of such intake,^9^ whereas the others did not.^8, 9, 10^ As for n-3 PUFA levels in blood, one of these Japanese studies also measured the plasma level of n-3 PUFAs and found that lower plasma DHA concentration was significantly associated with prenatal depressive symptoms (in the second trimester).^10^ Several studies conducted outside Japan have investigated the relationship between n-3 PUFA level in blood and depression in perinatal periods,^11, 12, 13, 14, 15^ but the results have not clarified the situation as yet.

Japan is well known for its high consumption of fish and seafood. According to National Health and Nutrition Surveys, however, consumption has declined over the past decade.^16, 17^ To date, no studies have reported on a possible relationship between n-3 PUFA levels in blood and psychological distress in early pregnancy. In this study, we therefore examined the possible relationship between serum n-3 PUFA levels and psychological distress among expectant mothers in early pregnancy.

## Materials and methods

### Study population

**Figure 1:**
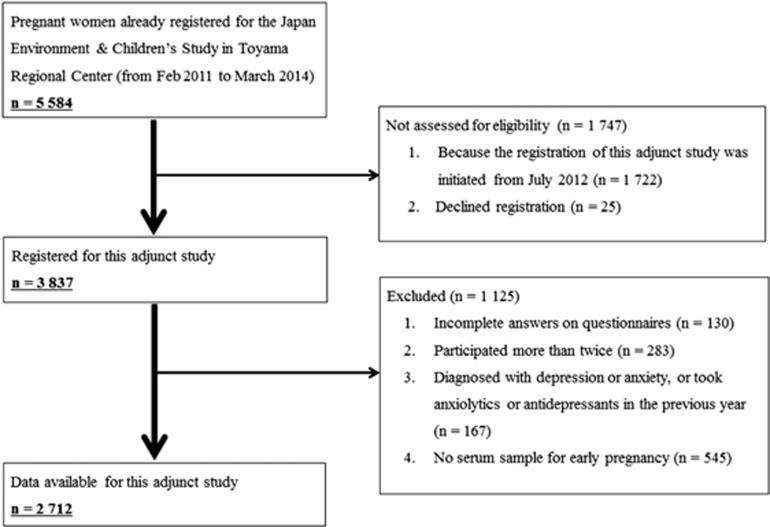
Flow diagram of the recruitment and exclusion process for pregnant women in the study.

This birth cohort study began in July 2012 at Toyama Regional Center as an adjunct study of the Japan Environment and Children's Study. The Japan Environment and Children's Study is a nation-based (government-funded) birth cohort study that is evaluating the impact of various environmental factors on children's health and development. It involves 103 106 parent–child pairs who were recruited from 15 areas in Japan from January 2011 to March 2014.^18^ Toyama prefecture is in Japan's Hokuriku region and 5584 parent–child pairs were recruited in the prefecture (registered from February 2011 to March 2014). In this prefecture, because we started the registration of this adjunct study from July 2012 (1.5 years later), 1722 pairs were not eligible for the study, and 25 participants declined to register for this adjunct study. Consequently, 3837 participants agreed to register for this study. As shown in Figure 1, after applying four exclusion criteria, a further 1125 subjects were excluded, leaving 2712 participants whose data were available for this specific study. From these 2712 participants, those with a Kessler Psychological Distress Scale (K6) score ⩾9 were assigned to the psychological distress group (*n=*283). From among the remaining study participants, we randomly selected 283 control subjects who met the matching criteria for age (within 1 year), educational background (three categories) and family income (three categories). Details of the categories are described in the section on data analysis and statistics. By estimating the sample size from a previous study,^11^ we determined that at least 55 case–control pairs were required to ensure adequate power to detect statistical difference in serum eicosapentaenoic acid (EPA) with an alpha level of 0.05 (two-tailed) and 80% power.

The protocol of this study was reviewed and approved by the Ministry of the Environment's Institutional Review Board on Epidemiological Studies and by the Ethics Committees of the University of Toyama. Written informed consent was obtained from all the participating women.

### Measurements

Self-administered questionnaires were collected during the first trimester (first questionnaire) and the second or third trimester (second questionnaire) and gathered data on demographic factors, physical and mental health, lifestyle, occupation, environmental exposure, habitation and socioeconomic status among other factors.^19^ Information was also obtained from the medical records on gravidity, related complications, parity, maternal anthropometry and other factors in the first trimester.^19^ We selected the following covariates: age, pre-pregnancy body mass index (BMI), smoking status, occupation, physical activity, marital status, pregnancy-associated nausea and medication as reported in the first questionnaire; educational background, family income and alcohol intake as reported in the second questionnaire; and age (in case of missing data in the first questionnaire) and parity noted in the medical records for the first trimester.

The K6 was included in both the first and second questionnaires and the scale from the former was used for the present study. The K6 was developed by Kessler and colleagues^20^ and the Japanese version was validated by Furukawa and colleagues.^21^ The K6 is a very short screening instrument that has good precision in the 90th to 99th percentile range of the population distribution and consistent psychometric properties across major sociodemographic subsamples.^20^ The items in the K6 ask participants how frequently they experienced symptoms of psychological distress (for example, feeling so sad that nothing can cheer you up) during the past 30 days. Responses are recorded on a five-point scale (4=all the time, 3=most of the time, 2=some of the time, 1=a little of the time and 0=none of the time), for a score range of 0–24. We defined a K6 score ⩾9 as indicative of psychological distress, which was the cutoff used in previous studies.^22, 23^ A validation study-estimated sensitivity and specificity were 77.8 and 86.4 in the Japanese population when using this cutoff to identify subjects at high risk of mood or anxiety disorders, diagnosed according to the criteria of the Diagnostic and Statistical Manual of Mental Disorders, fourth edition.^24^ We also exploratorily used a higher cutoff of K6⩾13, indicating severe psychological distress,^25^ as this has been suggested the optimal cutoff to equalize false-positive and false-negative results.^26^ In this case, because the present study was originally designed to use the cutoff score of 8/9, the matching control group for the severe psychological distress group (K6⩾13) does not cover the score between 9 and 12, eventually resulting in a cutoff of K6⩽8 for the control group.

### Fatty acid analysis

#### Blood samples

Non-fasting blood samples were collected and after the biochemical measurements were made, the residual serum samples were kept at −80 °C until analysis. Seventy-five percent of the samples were collected at 9–14 weeks of gestation and the remaining 25% were collected after 15 weeks.

#### Lipid extraction and methylation

The transmethylation method developed by Lepage and Roy was used.^27^ Briefly, plasma (100 μl) and tricosanoate methyl ester (20 μg; 23:0 methyl ester) as an internal standard were added to a 13 mm × 100 mm screw cap test tube with 2 ml methanol/hexane (4:1, v/v) solvent containing 50 μg ml^−1^ butylhydroxytoluene as an aid to prevent lipid oxidation during the procedures. The sample tubes were placed on ice. Two hundred microliters of acetylchloride was added to each sample and vortexed. The air in the sample tubes was replaced with nitrogen, and the tubes were capped and heated for 1 h at 100 °C for lipid extraction and methylation. The sample tubes were then cooled rapidly, shaken with 5 ml of 6% potassium carbonate solution and centrifuged at 2200* g* at 4 °C for 15 min. The hexane layer (supernatant) was transferred to a micro vial for analysis by gas chromatography.

#### Gas chromatography

Fatty acid methyl esters were analyzed with an Agilent 7890 A gas chromatograph (Agilent Technologies, Santa Clara, CA, USA) equipped with a split injector and an Agilent 7693 ALS automatic liquid sampler, and were detected using a flame ionization detector. Agilent ChemStation software (Rev. B.04.01. SP1, Agilent Technologies) was used to control the instrument and collect the data. The column was a DB-FFAP (15 m × 0.10 mm inner diameter; 0.10-μm film thickness; J&W Scientific, Agilent Technologies). The detector and injector temperatures were set at 250 °C. The oven temperature program was initiated at 150 °C with a 0.25-min hold, and then ramped at 35 °C min^−1^ to 200 °C; then 8 °C min^−1^ to 225 °C with a 3.2-min hold; and finally ramped at 80 °C min^−1^ to 248 °C with a 14.7-min hold. Hydrogen was used as the carrier gas at a linear velocity of 56 cm s^−1^.^28^ A custom-mixed, 28-component, quantitative methyl ester standard containing compounds with 10–24 carbons and zero to six double bonds was used for assignment of retention times to ensure accurate quantification (Nu-Chek Prep 462, Elysian, MN, USA). Fatty acid content was expressed as a percentage of total peak area, which was shown to correspond to weight percent (below 5%) by the quantitative standard mixture. The internal standard was used to calculate fatty acid concentration in serum. The minimum value of quantification for fatty acid measurement was set to 0.01% of total fatty acids. Researchers (YO, AH and TM) measuring the fatty acids were blinded to group allocation (case vs control) until after the fatty acid data were determined.

### Data analysis and statistics

Smoking status was classified as never smoker, ex-smoker who stopped before learning of pregnancy, ex-smoker who stopped on learning of pregnancy or current smoker. Alcohol intake was classified as never drinker, ex-drinker who stopped before learning of pregnancy, ex-drinker who stopped on learning of pregnancy or current drinker. For the 11 instances of missing data for alcohol intake in the second questionnaire (second or third trimester), data from the first questionnaire (first trimester) were substituted. Because the first questionnaire offered only three categories for alcohol intake (never drank, ex-drinker, current drinker), we allocated ‘ex-drinker' as ‘ex-drinker who stopped on learning of pregnancy'. ‘Occupation' (working or not) was categorized as yes or no. Educational background was categorized as graduated from junior high or high school, graduated from junior/technical college or graduated from university/higher educational institution. For six subjects who did not respond about educational background, we matched each with a control subject who similarly did not respond. Family income was categorized into <4 million, 4 million–6 million or >6 million Japanese Yen per year. For 27 subjects who did not respond to this question, again we matched each with a control subject who similarly did not respond. Parity was categorized as 0 or ⩾1. We defined ‘medication' as any medicine taken (not including supplements) after learning of pregnancy (yes or no). Physical activity was defined as walking for at least 10 min continuously per week (yes or no). Marital status was categorized as married (including *de facto* relationship), never married or divorced. Data were missing for height in one instance and for weight in five instances in the first questionnaire (first trimester) and data from the medical records were substituted. Age and pre-pregnancy BMI was used as a continuous variable. For the missing data that we could not substitute, we added the extra category of missingness for the variable for imputation. These imputations were used only for adjustment in regression analysis.

Data are expressed as means±s.d. or median (0.25, 0.75) unless stated otherwise. In descriptive analyses, differences in categorical and continuous variables among cases and controls were tested using the chi-square test and *t*-test, respectively. The Komogorov–Smirnov statistics showed that the fatty acids were not normally distributed, so the confidence band (0.25, 0.75) is shown and the Mann–Whitney *U*-test was used for analyzing these data.

To estimate the risk of psychological distress for each plasma fatty acid level, we categorized the participants according to the tertile distributions of fatty acid levels in controls. We then performed logistic regression analysis to calculate crude odds ratios (ORs) and 95% confidence intervals. Multiple logistic regression analysis was used to control for the following potential confounding factors: parity, smoking status, alcohol intake, pre-pregnancy BMI, medication, occupation, physical activity, marital status and having pregnancy-associated nausea. Tests for trend involved assigning categorical numbers in their fatty acid tertile and evaluating this as a continuous variable. Two-sided *P*-values less than 0.05 were considered to indicate statistical significance. Data were analyzed using statistical software, SPSS version 19.0 (IBM Japan, Tokyo, Japan).

## Results

Baseline characteristics of cases and controls are shown in Table 1. Besides the matching factors (age, educational background and family income), no significant differences were seen between the two groups in relation to smoking status, pre-pregnancy BMI, occupation, parity, physical activity, marital status, alcohol intake and medication. A significant difference was seen, however, for pregnancy-associated nausea; women with psychological distress had a higher rate than those without.

Table 2 shows comparisons of the serum fatty acid composition. Women with psychological distress were more likely to have lower levels of stearic acid (18:0). As for PUFAs, women with psychological distress had lower levels of EPA (20:5 n-3), docosapentaenoic acid (22:5 n-3), and total n-3 PUFAs than controls. DHA did not differ significantly between the two groups. Mean±s.d. data are included in Supplementary Table 1.

Table 3 shows the crude and multivariable ORs for psychological distress and 95% confidence intervals according to the tertiles for n-3 PUFAs. EPA was the only fatty acid in the crude models that showed significant ORs in the highest tertile compared with the lowest tertile. The trend test for this fatty acid was also significant. Furthermore, multivariable ORs that were adjusted for nine confounding factors (parity, smoking status, alcohol intake, pre-pregnancy BMI, medication, occupation, physical activity, marital status and pregnancy-associated nausea) also showed significance either in ORs of the highest vs the lowest tertile or in the trend tests. Stearic acid (18:0) showed no significant ORs in the highest tertile compared with the lowest.

When the higher cutoff score of K6⩾13 was applied, the case–control pairs were reduced to 74. The ORs and 95% confidence interval for psychological distress after adjusting for the nine confounding factors were 0.95 (0.97–2.475) for the second level and 0.30 (0.10–0.90) for the third level of EPA (*P*=0.04 for trend; Supplementary Table 2).

## Discussion

To our knowledge, this is the first study to report associations between serum n-3 PUFAs and risk of psychological distress in early pregnancy. Trend testing showed that serum EPA, but not docosapentaenoic acid and DHA, had significant inverse associations with ORs for psychological distress. We also found that women with psychological distress had a higher rate of pregnancy-associated nausea. This relationship persisted after adjusting for the nausea and other factors as covariates, indicating that lower EPA was independently associated with psychological distress.

In regard to the dietary intake of fish and/or n-3 fatty acids in Japan, Miyake *et al.*^8^ were the first to examine the risk of postpartum depression in a large-scale prospective study conducted in Osaka; however, no clear inverse associations were found. Later, the same group conducted a cross-sectional study of pregnant Japanese women in Kyushu and Okinawa (mean gestation, 18.5 weeks) and found that higher intake levels of fish, EPA and DHA were independently associated with a lower prevalence of depressive symptoms.^9^ Recently, when Shiraishi *et al.*^10^ examined the dietary intake of EPA and DHA in another cross-sectional study of pregnant Japanese women at an urban university hospital in Tokyo, they found no beneficial effects of n-3 PUFA intake on depressive symptoms.^10^ It is not clear why some studies have shown beneficial effects of n-3 PUFA intake on depressive symptoms, whereas other studies have shown no effects. Reasons could include different sample sizes, different periods when depressive symptoms were assessed (prenatal vs postpartum), the screening tools used and the study location (urban vs rural).

In regard to n-3 PUFA levels in tissues (blood), to date, among the Japanese studies mentioned above, only the most recent one by Shiraishi *et al.*^10^ also investigated the relationship between serum n-3 PUFA levels and depressive symptoms in pregnant Japanese women in the second trimester. They found that lower plasma concentration of DHA, but not EPA, was significantly associated with prenatal depressive symptoms.^10^ However, as the authors discussed, the largest limitation of their study was statistical power. Only 19 participants (5.8%) of 329 had depressive symptoms, making it impossible to adjust for other factors potentially related to depressive symptoms in their analysis. A rather small study from Belgium reported that levels of n-3 PUFAs in serum phospholipids and cholesteryl esters after delivery were significantly lower in the group of mothers who developed postpartum depression than in the group who did not.^11^ A study from Norway found a negative association between erythrocyte DHA and docosapentaenoic acid in late pregnancy and depression score at 3 months postpartum,^12^ while another from the United Kingdom found a weak positive association between erythrocyte EPA and perinatal onset depression.^13^ A large study from Australia found slight associations of lower erythrocyte n-3 PUFAs, lower erythrocyte EPA and higher erythrocyte omega-6 with postnatal depression determined by the Edinburgh Postnatal Depression Scale but not with postnatal depression diagnosed according to DSM criteria.^14^ A recent large study reported from Singapore revealed that plasma long-chain PUFA levels measured at 26–28 weeks of gestation were not associated with depression during the same period or at 3 months postpartum.^15^ Again, there is no clear explanation for these discrepancies. Perhaps, besides the factors of assessment period, screening tools and study location mentioned above, the part of the blood measured could be another factor to consider, given that erythrocytes usually show a longer turn-over of fatty acids than serum and plasma do.^29^

Interestingly, in this study, EPA had a stronger effect on psychological distress than DHA did. Several meta-analyses of clinical trials revealed that EPA is more effective for depression^30^ than DHA is. When meta-analysis is restricted to perinatal depression (seven trials),^31^ n-3 supplementation is not significantly more effective than placebo. The only study reporting positive findings^32^ used 2.2 g of EPA and 1.2 g of DHA daily (the highest ratio of EPA), suggesting that a high dose of EPA might also be important in perinatal depression. Why EPA is more effective for depression than DHA is not clear. One plausible explanation is that EPA, but not DHA, is an important substrate for cyclooxygenase and can compete with arachidonic acid, which produces pro-inflammatory eicosanoids,^33^ and depression is known to be associated with neuroinflammation.^34^

The pathophysiology of lower serum n-3 PUFAs associated with depressive symptoms in pregnant Japanese women is not fully understood. In rats, Olsson *et al.*^35^ reported that a diet low in n-3 fatty acids decreased serotonin and 5-hydroxyindole acetic acid concentrations, while Delion *et al.*^36^ found an association between n-3 fatty acid deficiency and significant elevations in cortical serotonin 5-HT2A receptor binding density. In a postmortem brain study, McKeith *et al.*^37^ suggested an increase in 5-HT2 receptor binding in major affective disorder as a possible pathophysiological mechanism.

The strengths of this study were that it was conducted in early pregnancy, a period not reported on previously, and it used the K6 Scale, which has been validated for use in normal populations for the screening of psychological distress and is widely used in Japan. In addition, the sample size was large enough to avoid a lack of statistical power.

The study also has several limitations. First, because of its cross-sectional design, we were not able to clarify causality. Second, the results of the study may not reflect the situation for all Japanese pregnant women because the sample was drawn from one prefecture of Japan only. Third, because high serum n-3 PUFA levels reflect regular fish consumption, which is perhaps a global marker of a healthy and varied diet, unmeasured residual factors might have confounded the results. Fourth, we excluded pregnant women who were diagnosed with depression or anxiety or who took anxiolytics or antidepressants (*n=*167). This exclusion might have weakened the association between serum n-3 PUFA levels and the prevalence of psychological distress because a meta-analysis has shown that studies using DSM-defined major depressive disorder found larger differences in serum n-3 PUFA levels than studies not using DSM criteria.^38^ This is supported by the fact that ORs were smaller in the present study when using the cutoff score of 13 than when using the cutoff score of 9 (0.30 vs 0.45). Last, we measured fatty acid composition of serum total lipids, 50% of which were triglycerides. Triglycerides have been reported to represent dietary intake from preceding days.^29^ Furthermore, because the samples were collected in a non-fasted state and no information was available on meals before the blood draw (which is known to have more effect on the biological variability of fatty acids in serum than in erythrocytes^39^), this might also confound the relationship between serum n-3 PUFA levels and the prevalence of psychological distress. If we had used erythrocyte membranes for fatty acid analysis, which show a longer turn-over than serum does, we might have seen a stronger association.

In conclusion, we found that EPA was inversely associated with the risk of psychological distress in early pregnancy. Although the higher rate of pregnancy-associated nausea was prevalent in women with psychological distress, adjusting for this factor as a covariate did not change these associations. The causality of these associations should be verified in further research, such as cohort studies and/or intervention studies.

## Figures and Tables

**Table 1:** Characteristics of study participants

	*Cases (*n=*283)*	*Controls (*n=*283)*	
Age (year)	31.5±5.0	31.5±4.9	Matching
*Educational background*			Matching
Graduated from junior high or high school	76	76	
Graduated from junior/technical college	135	135	
Graduated form university/higher educational institution	66	66	
			
*Family income (Japanese Yen per year)*			Matching	
<4 million	80	80		
4 million–6 million	93	93		
>6 million	83	83		
			
*Smoking status*			*P*=0.40	
Never smoker	168	186		
Ex-smoker who stopped before learning of pregnancy	62	54		
Ex-smoker who stopped on learning of pregnancy	45	37		
Current smoker	8	5		
			
Pre-pregnancy body mass index	20.8±3.0	20.8±3.1	*P*=0.99	
Occupation (yes/no)	217/61	216/59	*P*=0.89	
Parity of ⩾1 (yes/no)	131/152	135/148	*P*=0.74	
Physical activity (yes/no)	210/69	202/80	*P*=0.33	
Marital status			*P*=0.70	
Married (including de facto)	271	271		
Never married	7	10		
Divorced	3	2		
			
*Alcohol intake*			*P*=0.26	
Never drinker	89	106		
Ex-drinker who stopped before learning of pregnancy	37	42		
Ex-drinker who stopped on learning of pregnancy	150	130		
Current drinker	7	4		
			
*Pregnancy-associated nausea*			*P*=0.0002	
Never	21	45		
Only nausea	109	120		
Suffering from vomiting but able to eat	89	83		
Not able to eat	60	30		
			
Taking medication (yes/no)	78/205	68/215	*P*=0.52	

*P*-value: chi-square test for categorical variables and *t*-test for continuous variables.

**Table 2:** Serum fatty acid compositions of cases and controls

		*Cases (*n=*283)*		*Controls (*n=*283)*		P-*value*
		*Median*	*(0.25, 0.75)*	*Median*	*(0.25, 0.75)*	
*Saturated fatty acids*						
Myristic acid	14:0	0.90	(0.67, 1.23)	0.88	(0.65, 1.23)	0.84
Palmitic acid	16:0	22.51	(20.82, 24.57)	22.57	(20.70, 24.98)	0.20
Stearic acid	18:0	6.90	(6.22, 7.54)	6.99	(6.34, 7.80)	0.045
Arachidic acid	20:0	0.30	(0.25, 0.35)	0.31	(0.26, 0.35)	0.30
Behenic acid	22:0	0.67	(0.57, 0.79)	0.70	(0.59, 0.81)	0.12
Lignoceric acid	24:0	0.55	(0.45, 0.65)	0.56	(0.47, 0.67)	0.15
Total saturated fatty acids		34.49	(32.88, 36.41)	34.97	(32.87, 37.03)	0.11
						
*Mono-unsaturated fatty acids*						
Palmitoleic acid	16:1 n-7	1.40	(1.12, 1.84)	1.32	(1.08, 1.77)	0.08
Hexadecenoic	16:1 n-9	0.68	(0.49, 0.86)	0.65	(0.45, 0.84)	0.29
Vaccenic acid	18:1 n-7	1.79	(1.62, 2.00)	1.76	(1.60, 1.90)	0.07
Oleic acid	18:1 n-9	16.95	(15.31, 18.61)	16.78	(15.16, 18.68)	0.52
Gondoic acid	20:1 n-9	0.21	(0.17, 0.26)	0.21	(0.18, 0.26)	0.65
Erucic acid	22:1 n-9	0.79	(0.00, 1.31)	0.74	(0.00, 1.26)	0.27
Nervonic acid	24:1 n-9	1.56	(1.35, 1.80)	1.59	(1.42, 1.80)	0.17
Total mono-unsaturated fatty acids		23.75	(22.23, 25.42)	23.49	(21.76, 25.16)	0.11
						
*n-6 polyunsaturated fatty acids*						
Linoleic acid	18:2 n-6	20.30	(17.58, 23.30)	20.70	(17.77, 23.22)	0.49
Eicosadienoic acid	20:2 n-6	0.25	(0.21, 0.29)	0.26	(0.22, 0.29)	0.42
Dihomo-γ-linolenic acid	20:3 n-6	1.19	(0.89, 1.42)	1.17	(0.96, 1.43)	0.32
Arachidonic acid	20:4 n-6	5.80	(5.10, 6.54)	5.86	(5.21, 6.58)	0.22
Total n-6 polyunsaturated fatty acids		28.02	(24.75, 30.96)	28.24	(25.99, 31.33)	0.34
						
*n-3 polyunsaturated fatty acids*						
α-Linolenic acid	18:3 n-3	0.64	(0.51, 0.81)	0.65	(0.52, 0.82)	0.81
Eicosapentaenoic acid	20:5 n-3	0.53	(0.41, 0.79)	0.62	(0.46, 0.94)	0.001
Docosapentaenoic acid	22:5 n-3	0.42	(0.33, 0.53)	0.45	(0.37, 0.56)	0.02
Docosahexaenoic acid	22:6 n-3	3.39	(2.73, 4.06)	3.56	(2.95, 4.09)	0.09
Total n-3 polyunsaturated fatty acids		5.26	(4.34, 6.15)	5.50	(4.57, 6.43)	0.045

n-6/n-3		5.29	(4.49, 6.44)	5.25	(4.37, 6.19)	0.26

*P*-value: Mann–Whitney *U*-test.

**Table 3:** Odds ratios (95% confidence intervals) for psychological distress (K6 score ⩾9) according to tertile of n-3 polyunsaturated fatty acids in women in early pregnancy

	*Tertile of fatty acids*			P *for trend*
	*1 (Low)*	*2*	*3 (High)*	
*Eicosapentaenoic acid 20:5 n-3*				
Range	<0.518	0.518–0.832	>0.832	
Case	133	86	64	
Control	95	94	94	
Model 1^a^	1.00	0.65 (0.44–0.97)	0.49 (0.32–0.73)	0.0005
Model 2^b^	1.00	0.67 (0.44–1.02)	0.47 (0.30–0.73)	0.0007
				
*Docosapentaenoic acid 22:5 n-3*				
Range	<0.399	0.399–0.518	>0.518	
Case	112	93	78	
Control	95	94	94	
Model 1^a^	1.00	0.84 (0.56–1.25)	0.70 (0.47–1.06)	0.09
Model 2^b^	1.00	0.91 (0.60–1.37)	0.73 (0.48–1.13)	0.16
				
*Docosahexaenoic acid 22:6 n-3*				
Range	<3.13	3.13–3.92	>3.92	
Case	103	97	83	
Control	95	94	94	
Model 1^a^	1.00	0.95 (0.64–1.42)	0.81 (0.54–1.22)	0.9
Model 2^b^	1.00	0.97 (0.64–1.47)	0.79 (0.52–1.22)	0.29

a Crude data.

b Multivariable models were adjusted for parity, smoking status, alcohol intake, body mass index, medication, occupation, physical activity, marital status and having pregnancy-associated nausea.
